# Neuroimaging based biotypes for precision diagnosis and prognosis in cancer-related cognitive impairment

**DOI:** 10.3389/fmed.2023.1199605

**Published:** 2023-08-29

**Authors:** Shelli R. Kesler, Ashley M. Henneghan, Sarah Prinsloo, Oxana Palesh, Max Wintermark

**Affiliations:** ^1^Division of Adult Health, School of Nursing, The University of Texas at Austin, Austin, TX, United States; ^2^Department of Diagnostic Medicine, Dell School of Medicine, The University of Texas at Austin, Austin, TX, United States; ^3^Department of Oncology, Dell School of Medicine, The University of Texas at Austin, Austin, TX, United States; ^4^Department of Neurosurgery, The University of Texas MD Anderson Cancer Center, Houston, TX, United States; ^5^Department of Psychiatry, Virginia Commonwealth University, Richmond, VA, United States; ^6^Department of Neuroradiology, The University of Texas MD Anderson Cancer, Houston, TX, United States

**Keywords:** cancer, cognition, neuroimaging, biotypes, precision medicine

## Abstract

Cancer related cognitive impairment (CRCI) is commonly associated with cancer and its treatments, yet the present binary diagnostic approach fails to capture the full spectrum of this syndrome. Cognitive function is highly complex and exists on a continuum that is poorly characterized by dichotomous categories. Advanced statistical methodologies applied to symptom assessments have demonstrated that there are multiple subclasses of CRCI. However, studies suggest that relying on symptom assessments alone may fail to account for significant differences in the neural mechanisms that underlie a specific cognitive phenotype. Treatment plans that address the specific physiologic mechanisms involved in an individual patient’s condition is the heart of precision medicine. In this narrative review, we discuss how biotyping, a precision medicine framework being utilized in other mental disorders, could be applied to CRCI. Specifically, we discuss how neuroimaging can be used to determine biotypes of CRCI, which allow for increased precision in prediction and diagnosis of CRCI *via* biologic mechanistic data. Biotypes may also provide more precise clinical endpoints for intervention trials. Biotyping could be made more feasible with proxy imaging technologies or liquid biomarkers. Large cross-sectional phenotyping studies are needed in addition to evaluation of longitudinal trajectories, and data sharing/pooling is highly feasible with currently available digital infrastructures.

## Introduction

Up to 75% of survivors of cancers that originate outside of the central nervous system demonstrate cognitive impairment before, during or following cancer treatments (1–4). CRCI typically presents as difficulties with attention, executive function, memory, and processing speed that can last months or years after the conclusion of treatment (1–5). CRCI can have debilitating effects on survivors’ quality of life including their social and occupational functioning (3, 4, 6–9), and is associated with increased death including cancer-related and all-cause mortality (10–12). Millions of cancer survivors [16.9 million in 2019 alone (13)] are at risk for CRCI.

Cognitive symptoms vary greatly from person to person in terms of domains impacted, as well as symptom severity and trajectory (e.g., late onset, persistent, improving over time). In fact, most symptoms associated with treatment toxicities or cancer pathology are highly variable and unique to each survivor. Thus, recent calls to action have been made for *precision survivorship* in the field of oncology, where some genetic risks for treatment-related toxicities have been identified in pediatric oncology populations (14). Since 2016, the National Cancer Institute (NCI), has prioritized *precision medicine* which includes “precision oncology” and “precision survivorship” (15).

The NCI defines precision medicine as using a person’s own biology to diagnose cancer or determine a course of treatment for cancer. Many advancements have been made in precision medicine with respect to cancer diagnostics and treatments, but there has been a lag in applying precision medicine to cancer survivorship, especially for adult cancer survivors (16). Pre-treatment data could be used to determine who is most susceptible to treatment related toxicities, including CRCI.

Precision medicine approaches to CRCI may significantly improve the diagnosis and prognosis of this syndrome. Under or over diagnosing of CRCI incurs significant costs such as negative individual, familial (including impact to the caregiver), and societal burden. The economic benefits of accurate and timely diagnosis have been shown to significantly reduce health care costs. For example, one study showed that health care costs would be reduced by more than half to two-thirds if a diagnosis had been made earlier (17). Patients with CRCI often experience increased stress, anxiety, and depression, loss of employment opportunities, and more social isolation. Patients’ distress can be significantly reduced by health-related perceptions of situational control, validation, and course of deficit, all of which are impossible without a correct diagnosis. Understanding and correctly characterizing the CRCI condition is also imperative for precisely developing and guiding patients toward interventions that can ameliorate their symptoms.

## Defining CRCI

Clinical symptoms of CRCI are currently assessed using standardized neuropsychological tests and/or self-report measures. Guidelines for using neuropsychological tests in CRCI research were published in 2011 (18). These tests provide insight regarding an individual’s cognitive strengths and weaknesses and can assist with diagnosing neuropsychiatric conditions. However, several reports have suggested that these tests may have limited sensitivity, specificity, and reliability for CRCI (19–22). Many of these measures were developed to assess severe neuropathology (21, 23) and therefore may have ceiling effects for CRCI. These tests tend to lack ecological validity (24, 25) and there is also evidence of ethnic or racial biases in standardized neuropsychological testing (26–28).

Guidelines for using self-report measures in CRCI research were published in 2021 (29). Self-report measures reflect daily functioning (30), correlate with mood and psychological factors (24, 30–32), and some were developed specifically for cancer survivor populations (33, 34). Incidences of CRCI are higher when subjective measures are used (3) suggesting they may be more sensitive to this syndrome. However, self-report measures also have several limitations including susceptibility to retrospective recall, response, and state-dependent biases as well as demand characteristics (35–38). Importantly, some studies have found elevated response bias among cancer survivors compared to normative groups (36, 37, 39). Self-reported CRCI has been criticized for rarely correlating with neuropsychological performance (31). However, it is likely that self-report and neuropsychological tests measure different aspects of CRCI and cannot be considered interchangeable (40). Accordingly, evidence suggests that subjective and objective measures of CRCI represent different neural phenotypes. Both objective and subjective measures of CRCI have been shown to correlate with structural and functional brain abnormalities, but these appear to be largely distinct and non-overlapping (41–45).

Neither standardized cognitive tests nor self-report measures can track an individual’s day-to-day variability in cognitive function. Both rely on population-based methods (between group variability) rather than precision-based methods (within person variability). Cognitive functioning is subject to individual variability and real-life demands, thus using one’s own cognitive variability within one’s own environment may be a more ecologically valid and unbiased way of assessing CRCI. A precision health approach to defining CRCI, which is an individually focused approach, is especially beneficial for historically marginalized groups, such as racial/ethnic minorities, as mentioned above.

## Dichotomous definitions of CRCI

One of the greatest challenges to clinical management of CRCI is its inconsistent definition within the existing literature. As noted above, CRCI is currently evaluated using neuropsychological test performance. However, these tests rarely provide normative cutoff scores for deficient performance. Therefore, classification of impairment based on these tests in patients with cancer has relied on arbitrary cutoff scores leading to inconsistent results (46). To better harmonize CRCI studies, the International Cognition and Cancer Task Force (ICCTF) suggested a specific metric for determining impairment based on z-scores (18). Few studies have compared the ICCTF definition to other rubrics, although one study showed it was nearly twice as sensitive to impairment compared to other definitions (47). However, different results occur depending on the reference group used for z-score calculation and the size and composition of the testing battery (48, 49).

A major limitation of the z-score and similar approaches, including standardized based regression (50) and reliable change index (22), is that they result in dichotomous classification (yes/not impaired; yes/no declined). Dichotomous classification is not consistent with the complex, continuous nature of brain function. Akin to “black and white thinking,” dichotomization ignores dimensional nuances that could provide greater insight regarding cognitive function (51). In fact, a recent study demonstrated that binary impairment definitions, including the ICCTF criterion, yield unreliable cognitive classifications (52). This is particularly important for CRCI given the historical controversy regarding its existence. Specifically, many patients are classified as “unimpaired” on neuropsychological tests despite experiencing significant difficulties completing everyday cognitive tasks. This inconsistency may suggest that there are latent subgroups of patients whose cognitive changes do not fit into black and white categories.

Several statistical techniques exist that can determine subgroups within the data that are not readily apparent (Table 1). For example, growth mixture modeling, latent profile analyses and clustering have been used to discover CRCI subgroups in a data-driven manner. Growth mixture modeling determines latent subgroups based on different trajectories of an outcome across longitudinal timepoints (69). Prior studies of CRCI using growth mixture modeling have identified up to five latent subgroups of self-reported or objective cognitive function, with the most common finding being three subclasses (53–58).

**Table 1:** Summary of studies utilizing precision health methods for subtyping CRCI.

References	Sample	Subtyping method	Cognitive outcome(s)	CRCI subtypes identified
Allemann-Su et al. (53)	*N* = 397 adult breast cancer	Growth mixture modeling	AFI	3 (High, moderate, low effective action) 4 (Very low, low, moderate, high attentional lapses) 2 (Low and high interpersonal effectiveness)
Merriman et al. (54)	*N* = 209 adult breast cancer *N* = 122 adult noncancer controls	Growth mixture modeling	PAOFI	3 (More frequent, persistent, almost never)
Morin and Midlarsky (55)	*N* = 403 adult cancer	Growth mixture modeling	Total recall	3 (High, middle, low recall)
Rolfe et al. (56)	*N* = 130 adult breast cancer	Growth mixture modeling, K-means Clustering	AVLT Trials 1–5, 7, & 8; NART	3 Classes immediate retention; 2 classes delayed recall; 3 classes learning
Tometich et al. (57)	*N* = 319 adult breast cancer *N* = 347 adult noncancer controls	Growth mixture modeling	NAB digits forward, digits backward, list learning immediate recall, short delay, long delay; COWA; TMT-A and B; Digit Symbol Coding test; Logical Memory I & II; FACT-Cog	2 (High and low symptom)
Westrick et al. (58)	*N* = 2,986 adult cancer	Growth mixture modeling	Immediate and delayed recall of a 10-word list; IQCODE	5 (Very low, low, medium-low, medium-high, high memory loss)
Agelink van Rentergem et al. (59)	*N* = 62 adult breast cancer *N* = 228 adult noncancer controls	Latent profile analysis, K-means clustering, Hierarchical Clustering	ACS	2 (Cognitively unaffected, Cognitively affected)
Atallah et al. (60)	*N* = 1,329 adult cancers (breast, gastrointestinal, gynecological, or lung cancer)	Latent profile analysis	AFI	3 (High, moderate, low attentional function)
Karlson et al. (61)	*N* = 101 pediatric cancers (leukemias, lymphoma, solid tumors, brain tumors and other cancers)	Latent profile analysis	WISC-IV, Digit Span Forward/Backwards, Letter-Number Sequencing, Processing Speed; WASI/WAIS- III, Digit Span Forward/Backwards, Letter-Number Sequencing, Processing Speed; WIAT-II, Stroop Color and Word Test, CPT-II; CVLT-C/CVLT-II	3 (Class 1, 2, 3)
Peterson et al. (62)	*N* = 364 pediatric cancers (brain tumors, acute lymphoblastic leukemia) or ADHD	Latent profile analysis	SCT	3 (Low, medium, high SCT)
Sharkey et al. (63)	*N* = 89 pediatric cancer (brain tumor)	Latent profile analysis	WISC-IV/WISC-V, PSI, Digit Span; TOL-DX	4 (Average, Cognitive Deficit, Social/Cognitive Deficit, Discrepant)
Utne et al. (64)	*N* = 365 adult cancers (breast, gastrointestinal, gynecological, or lung)	Latent profile analysis	AFI	3 (Low, moderate, high function)
Li et al. (65)	*N* = 132 adult breast cancer *N* = 45 adult noncancer controls	Latent General Linear Model Analysis	MASQ; CPT, Vigilance, Distractibility; D-KEFS CWI, Inhibition, Word Reading, Color Naming; D-KEFS TMT, Motor Speed, N/L Switching, Letter Sequencing, Number Sequencing, Visual Scanning; D-KEFS VFT, Letter, Category; WAIS-III, Digit Symbol Coding; PASAT, 3″, 2″; WMS-III, Faces I, Faces II; CVLT-2, Trial 1, MRR, List B, Trials 1–5, LDFR; GPT, Right & Left	N/A
Romero-Garcia et al. (66)	*N* = 17 adult cancer (diffuse glioma) *N* = 268 adult noncancer controls	K-means clustering	WAIS-IV; BMIPB; OCS	4 (Neuropsychology Memory, Verbal Skills and Attention, OCS-Bridge Attention, Nonverbal Skills and Low Variance)
Kesler et al. (67)	*N* = 80 adult breast cancer *N* = 103 adult noncancer controls	Biotypes (k-means clustering and random forest proximity of neuroimaging data)	CTMT Trials 1 & 5; D-KEFS-VFT; RAVLT, Immediate Recall, Delayed Recall; BRIEF-A	3 (Low cognitive function, Cognitively resilient, Moderately low cognitive function)
Mulholland et al. (68)	*N* = 80 adult breast cancer *N* = 82 adult noncancer controls	Biotypes (k-means clustering and random forest proximity of neuroimaging data)	CTMT Trials 1 & 5; D-KEFS-VFT; RAVLT, Immediate Recall, Delayed Recall; BRIEF-A	3 (Low cognitive function, Cognitively resilient, Moderately low cognitive function)

Neuropsychological measures: ACS, Amsterdam Cognition Scan; AFI, Attentional Functional Index; AVLT, Auditory Verbal Learning Test; BMIPB, Brain Injury Rehabilitation Trust Memory and Information Processing Battery; BRIEF-A, Behavioral Rating Inventory of Executive Function - Adult Version; COWA, Controlled Oral Word Association Test; CPT, Conners’ Continuous Performance Test; CPT-II, Conners’ Continuous Performance Test-Second Edition; CTMT, Comprehensive Trail Making Test; CVLT-C, California Verbal Learning Test for Children; CVLT-II, California Verbal Learning Test- Second Edition; D-KEFS, Delis-Kaplan Executive Function System; FACT-Cog, Functional Assessment of Cancer Therapy-Cognitive Function; GPT, Grooved Pegboard Test; IQCODE, Jorm Informant Questionnaire for Cognitive Decline; MASQ, Multiple Ability Self-Report Questionnaire; NAB, Neuropsychological Assessment Battery; NART, National Adult Reading Test; OCS, Oxford Cognitive Screen; PAOFI, Patient Assessment of Own Functioning Inventory; PASAT, Rao Paced Auditory Serial Addition Test; RAVLT, Rey Auditory Verbal Learning Test; SCT, Sluggish Cognitive Tempo Scale; TMT, Trail Making Test; WASI, Wechsler Abbreviated Scale of Intelligence; WAIS- III, Wechsler Adult Intelligence Scale-III; WAIS-IV, Weschler Adult Intelligence Scale IV; WIAT-II, Wechsler Individual Achievement Scale-II; WISC-IV, Wechsler Intelligence Scale for Children- Fourth Edition; WISC-V, Wechsler Intelligence Scale for Children-Fifth Edition; WMS-III, Wechsler Memory Scale-III; TOL-DX, Tower of London-Drexel Version. Neuropsychological measures subtest/subscales: CWI, Color Word Interference Test; VFT, Verbal Fluency Test; TMT, Trial Making Test; N/L Switching, Number-Letter Switching; MRR, Middle Region Recall; LDFR, Long Delay Free Recall.

Once the latent subgroups are identified, characteristics of the subgroups can be examined to determine their clinical significance including factors associated with risk and resilience. For example, using growth mixture modeling, Merriman et al. (54) identified three latent subgroups of subjective CRCI in patients with breast cancer. They labeled the subgroups “more frequent,” “persistent,” and “almost never” based on frequency of cognitive complaints. They found that patients in the “more frequent” subgroup had unique profiles of treatment regimens, genotypes, and psychiatric symptoms compared to the other subgroups (54). Alternatively, covariates can be included in the initial model to examine their impact on the longitudinal outcome (70).

Latent profile analysis also seeks to identify unknown subgroups within a larger population using a probabilistic model that describes the distribution of the data (71). Latent profile analysis is appropriate for cross-sectional and longitudinal data. Like growth mixture modeling, latent profile analysis allows for inclusion of covariates to predict latent subgroup membership. Latent profile analysis has been applied to study CRCI subgroups in both pediatric and adult cancers (59–64).

Other latent general linear model analysis techniques have been used to provide insight regarding the association of self-reported cognitive function and neuropsychological tests in patients with breast cancer (65). However, real-world data do not always meet linear model assumptions. Clustering is another statistical technique useful for discovering latent subgroups which is appropriate for linear or nonlinear cross-sectional or longitudinal data. Clustering is based on distances between data points and aims to identify subgroups within a sample that have both high within-group similarity and high between-group dissimilarity. Very few studies have applied clustering to examine profiles of CRCI. Romero and colleagues identified four subtypes of neuropsychological testing scores using k-means clustering (66). The authors interpreted these findings at the test level in terms of which tests were sensitive to impairment, but their results suggested subgroups of participants with different levels of impairment (66). Another study employed k-means clustering as a sensitivity analysis for their latent profile analysis, both of which identified only a dichotomous solution (59).

These studies represent advances in the literature towards a more precise definition of CRCI. However, the mechanisms that result in various symptoms must be known to develop treatments, particularly the mechanisms that differentiate patients who respond better to one treatment versus another. Mechanistic knowledge cannot be derived from symptom assessments alone given that similar symptoms can result from different pathologies (72). Neuropsychological tests, for example, can identify memory, attention, or other cognitive domain deficits. However, these tests summarize multiple cognitive processes into one score and thus are too broad for identifying the precise neural mechanisms involved. Novel data driven approaches as used in the studies above are more granular and able to classify patients into different subtypes based on behavioral data, but the resultant subtypes do not consider the differences in pathophysiology that would dictate precision treatment.

## CRCI is a brain-based disorder

The biologic mechanisms of cognitive disorders are frequently examined using neuroimaging techniques. Multiple magnetic resonance imaging (MRI) studies have demonstrated that CRCI is a brain-based syndrome characterized by significant changes in brain function and structure. Breast cancer survivors who receive chemotherapy treatment tend to show more pronounced global, cortical gray matter and subcortical white matter volume loss when compared with chemotherapy naïve and noncancer controls [see reviews by Sousa et al. (41), Schroyen et al. (42), and Niu et al. (73)]. McDonald et al. (74) reported the first prospective, longitudinal volumetric MRI study of CRCI. They observed a significant reduction over time in regional gray matter density from pre-chemotherapy to 1-month post-chemotherapy followed by partial improvement over time (74). Gray matter alterations have also been demonstrated in very long-term survivors (75–77). We uniquely used volumetric MRI to estimate cortical brain age and demonstrated that it is significantly increased from pre- to post- breast cancer chemotherapy (78). We also distinctively demonstrated that chemotherapy treated breast cancer survivors are significantly more likely to be classified as having incipient Alzheimer’s disease based on volumetric brain network organization compared to chemotherapy naïve survivors (79).

Pathologic white matter changes determined using T2-weighted MRI in patients with cancer exposed to chemotherapy were first described in the nineties (80–82). These changes tended to occur acutely after chemotherapy completion but persisted at 1 year of follow-up (81). Diffusion tensor imaging (DTI) has demonstrated abnormal white matter integrity in the corpus callosum of chemotherapy treated patients with breast cancer compared to noncancer controls (83). Subsequent studies have shown diffuse white matter integrity abnormalities that correlate with cognitive impairment (84, 85), indicating that a demyelinating process may partly underlie CRCI (75). DTI was also uniquely used by our group to computationally simulate the effects of aging on white matter organization, demonstrating that chemotherapy treated breast cancer survivors have lower resilience to brain aging compared to non-cancer controls (86).

Task-based functional MRI (fMRI) studies suggest that certain cognitive tasks are more challenging for patients with breast cancer as they demonstrate expanded recruitment of brain regions to maintain task accuracy. Several lines of research suggest that chemotherapy used in treatment of breast cancer upregulates neural activity (82, 87–91). Brain hypoactivation compared to non-cancer controls has also been reported in several cross-sectional studies (92, 93), yet brain hyperactivation is more often evident over time and is associated with self-reported cognitive complaints (90, 91, 94, 95).

Hyperactivation may therefore help explain the discrepancy that is commonly observed between neuropsychological tests and self-reports of cognitive function. Specifically, patients with breast cancer often demonstrate normal objective task performance but report significant cognitive difficulties (21, 96). Hyperactivation is believed to reflect neural compensation in response to brain injury, which can thus mask the underlying deficit (92). However, patient awareness of the additional neural effort required to maintain performance is reflected in their low self-ratings of cognitive function. Hyperactivation has been consistently observed in association with aging and is greater in individuals with age-related neurodegeneration (97–99).

Resting state fMRI functional connectivity studies provide insight regarding the brain network’s organization in terms of parallel information processing. Some studies show diffuse hyperconnectivity in chemotherapy exposed breast cancer patients and survivors compared to controls (88, 100, 101) while others have shown hypoconnectivity (45, 102–106). Given the brain’s vast complexity, the interpretation of hyper- vs. hypo- activation/connectivity is difficult especially since both can exist simultaneously. Most observations have been made by comparing patients and controls. It is likely that there are subgroups of patients characterized by distinct patterns of brain abnormalities which may help clarify some of the inconsistent findings observed across studies.

### Biotyping

Historically, neuropsychiatric conditions have been diagnosed and categorized based on the patient’s symptoms and the level of distress or functional impairment they experience. However, this approach is limited as it fails to capture the wide-ranging neurobiological mechanisms that can underlie similar symptomatology. This poses a significant challenge for the development of precision medicine, which requires a more nuanced understanding of the underlying mechanisms. To improve precision medicine, neuropsychiatric research is currently focusing on diagnoses that emphasize biology. For example, the National Institutes of Health’s Research Domain Criteria (RDoC) (107) and the Bipolar-Schizophrenia Network for Intermediate Phenotypes (B-SNIP) (108) projects aim to define subgroups of patients within a traditional diagnostic category based on neurobiologic profiles (107, 109).

The RDoC framework involves examination of psychological and biological dimensions across various levels of functioning within six primary functional domains. The RDoC framework emphasizes the measurement of behavioral, physiological, and self-reported data to gain a comprehensive understanding of each diagnostic dimension. The primary goal of the RDoC framework is to facilitate new research approaches that will enhance precision medicine for mental health conditions. B-SNIP is a multi-site consortium that aims to establish biotypes of mood and psychotic disorders (110, 111). Their work has resulted in a large, well-described neuropsychiatric population in which the RDoC framework can be applied. This includes examining different aspects of biotype expression such as demographics, symptom clusters, genetic variation, and treatment response.

**Figure 1:**
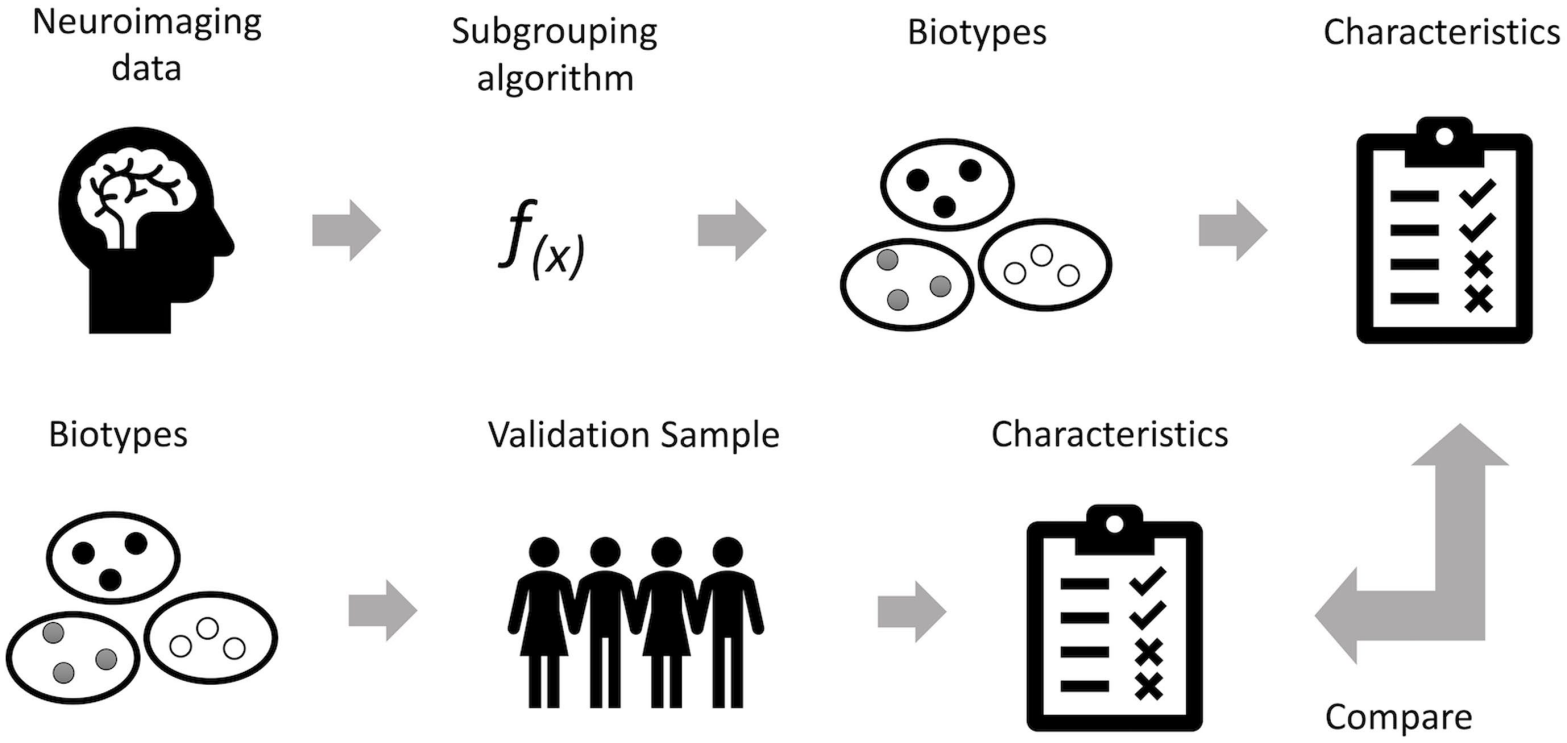
Overview of biotyping using neuroimaging. Neuroimaging can be used to determine biological subtypes (biotypes) of cancer-related cognitive impairment (CRCI). A subtyping algorithm, such as clustering, is applied to neuroimaging data (volumes, connectivity values, etc.). This results in distinct subtypes of patients who have common patterns of brain function or structure that may represent neural mechanisms of cognitive impairment. Behavioral or clinical characteristics, such as cognitive test performance, are then measured for each subgroup to determine the clinical relevance of the subgroups. Best practice is to then test the biotypes by applying the subtyping algorithm solution to an independent validation sample of patients, measuring the characteristics of interest and comparing these results to those from the original sample. Other validation methods include applying a naive algorithm to the data to determine if the same biotypes are replicated and examining longitudinal trajectories of different biotypes.

Machine learning techniques applied to neuroimaging data can be used to identify subgroups with distinct profiles of brain abnormalities (Figure 1). These biotypes represent disease subtypes based on their neural mechanisms. Most biotyping studies to date have relied on resting state functional magnetic fMRI. Resting state fMRI is relatively easy to acquire as it has no behavioral requirements, and it has consistently been shown across multiple studies to be highly sensitive to even subtle neuropathology (112). Resting state fMRI data are used to measure the connectivity of intrinsic functional brain networks (113). The sensitivity of these functional brain networks stems from various factors including their relatively high consumption of physiological resources, their association with gene expression patterns that are important for synaptic function and their involvement in multiple aspects of information processing (114–117).

Using machine learning and resting state fMRI data, Drysdale and colleagues identified four unique patterns of abnormal intrinsic functional brain connectivity in individuals with major depressive disorder (118). Each of the four biotypes presented with a unique profile of clinical features, highlighting the imprecision of the single diagnostic category used to describe them. Using similar methods, biotypes of other neuropsychiatric and neurological conditions have also been identified, including bipolar disorder, multiple sclerosis, Parkinson’s disease, attention deficit disorder, and schizophrenia (119–124).

To date, there have been few studies of CRCI biotypes. By applying clustering algorithms to resting state brain fMRI functional connectivity data, we previously identified three distinct profiles of abnormal brain connectivity, i.e., biotypes, in chemotherapy treated breast cancer survivors (67). Each biotype was associated with a unique cognitive phenotype as well as distinct demographic and clinical characteristics. We also demonstrated that our biotype algorithm was reproducible in an independent dataset. In a follow-up study (68), we demonstrated that each biotype had unique genetic and psychologic characteristics. We also showed that dichotomous, symptom-based classification combined patients with *distinct profiles of abnormal brain connectivity* into a single category of impaired cognition (68). In other words, divergent biological mechanisms were ignored by symptom assessment. Additionally, symptom-based assessment was unable to distinguish cognitive, genetic, or psychologic characteristics. Further research is needed to determine the reliability of biotypes across cancer types, if biotypes can predict different CRCI trajectories, and importantly if biotypes are associated with different responses to CRCI interventions.

### Prediction of outcomes

Another critical barrier to appropriate clinical management of CRCI is the inability to determine which patients will experience this adverse event. Several studies have revealed correlations between CRCI and older age, lower physical and mental activity (i.e., cognitive reserve), higher disease severity and greater chemotherapy treatment intensity (125–127). As described above, neuroimaging studies indicate that the final common biologic pathway of cancer-related cognitive impairment is altered brain structure and function (4, 128, 129). Several studies have shown that baseline neuroimaging can be used to build models that are accurately predictive of future cognitive outcomes. For example, baseline quantitative MRI has been used previously to accurately predict the onset of neurogenerative disorders, post-neurosurgical cognitive status, and cognitive rehabilitation outcome (130–134).

Although traditional inferential statistical approaches can be valuable in this space, machine learning algorithms (135) are more commonly used to build these predictive models because of the high dimensional and often nonlinear nature of quantitative neuroimaging data. Additionally, the priority for predictive models tends to be generalizability rather than inference and machine learning methods emphasize accuracy across samples and conditions. CRCI is a complex, heterogeneous condition with numerous potential contributors and confounds that must be considered in predictive models. The large number of potential demographic and clinical predictors in combination with the high dimensional nature of neuroimaging data warrants large sample sizes which are not always feasible. Compared to traditional statistical methods, machine learning algorithms are often better at handling this “large p, small n” problem (136). Several studies have demonstrated that the inclusion of quantitative neuroimaging metrics significantly improves the accuracy of machine learning predictive models for cognitive outcomes (137–139).

Some studies have shown cognitive impairment and brain injury in patients with breast cancer prior to chemotherapy treatment suggesting that tumor pathogenesis may contribute to CRCI (7, 140–146). These early abnormalities may reflect a vulnerable baseline condition within the brain that is later compounded by anti-cancer treatments, resulting in chronic CRCI. Our group pioneered the use of pre-treatment neuroimaging biomarkers to predict long-term CRCI following breast cancer treatment (147, 148) and other groups have since followed (149). However, this research has relied on binary cognitive outcomes or continuous outcomes with no definition of deficit. Thus, the identification of CRCI biotypes could increase the precision of these prediction algorithms.

As noted above, biotyping can also be used to predict intervention response. Given the complexity of neuropsychiatric disorders, several treatment regimens must often be tried to find the most effective one for a given patient. Interventions for CRCI are currently very limited but several show some promise, including cognitive training, cognitive rehabilitation, physical exercise, and neuromodulation (150–154). However, it is unknown which patients benefit most from which intervention. The traditional trial-and-error approach to prescribing interventions results in unnecessary side effects, time toxicity, increased costs, and delays in symptom management.

The overarching goal of biotyping is to identify disease subtypes that respond differently to various interventions such that precise treatment plans can be made for patients within that subtype. Drysdale et al. demonstrated different outcomes among depression biotypes after transcranial magnetic stimulation treatment (118). Specifically, using biotypes, they identified *a priori* which patients were most likely to benefit from the intervention. It is likely that patients with CRCI will not homogeneously respond to any one intervention and therefore biotypes could be useful in predicting treatment response. However, few if any CRCI intervention studies to date have included neuroimaging biomarkers.

## Interventions for CRCI

Once a precision health CRCI diagnosis or prognosis is made, an intervention may be required. Currently, there are no standardized, evidence-based interventions specifically for CRCI. However, several pharmacological and behavioral/integrative interventions have been examined as potential treatments for CRCI. Pharmaceutical treatments have included psychostimulants (155, 156), nicotine patches (157), anemia medications (158), and anti-dementia medications (159). Nguyen and Ehrlich described multiple other drugs that could potentially be repurposed for treating CRCI (160). Preclinical studies have evaluated several novel agents including PAN-811, a ribonucleotide reductase inhibitor (161), mesenchymal stem cells (162–164), functionalized mitochondria (165), dual leucine zipper kinase (166), histone deacetylase 6 inhibitor (167), KU-32, for mitochondrial repair (168), and astaxanthin, an antioxidant (169), among others. However, these treatments have yet to be translated into clinical studies.

There have also been several studies examining behavioral (e.g., psychoeducational, cognitive behavioral therapy, compensatory strategies, cognitive training, cognitive rehabilitation), lifestyle (e.g., physical activity, diet, stress management), neuromodulation (neurofeedback, transcranial magnetic stimulation), and integrative (e.g., acupuncture, music, meditation, yoga) interventions for CRCI (153, 170–176). A meta-analysis of 29 randomized control trials reported that the best options among behavioral interventions for CRCI in descending order of efficacy were: meditation/mindfulness-based stress reduction, combined cognitive training with exercise, cognitive training, cognitive rehabilitation, exercise, cognitive behavioral therapy, qigong, supportive therapy, yoga, and acupuncture (170). Another meta-analysis of nonpharmacological interventions for CRCI reported significant effects on objective measures of attention, immediate recall, and processing speed and subjective cognitive function, depending on intervention type and mode of delivery (177).

Measuring outcomes in CRCI intervention trials based on symptoms alone may yield inaccurate results given the limited sensitivity and specificity these assessments have for CRCI and the lack of reliable impairment cutoffs. For example, several previous CRCI intervention trials have observed few if any significant changes in cognitive testing scores yet demonstrated significant effects on functional brain metrics in breast cancer survivors (178, 179). Stimulant medication trials for CRCI have shown very mixed results (180) despite anecdotal evidence that these medications can be helpful for certain patients with CRCI and fatigue. It is possible that a subgroup of patients benefits from these medications or that the outcome measures used in these trials were not sufficiently sensitive for detecting cognitive changes. Biotypes allow us to identify biologically based classifications that could be used as clinical endpoints for intervention trials and to determine more accurate cut off scores for classifying impairment from symptom assessments.

## The role of animal models

Animal models are not likely to be specifically useful for biotyping or prediction modeling since these methods rely on the patient’s individual biology and developing animal models for CRCI poses significant challenges due to the intricate nature of replicating the human disease (181). However, for optimal clinical utility, CRCI diagnostic and prediction models require further knowledge regarding which chemotherapies are associated with cognitive deficits. The biological effects exerted by each chemotherapy drug are extensive, and this complexity increases when combined with other medications. This cannot be ethically examined in human patients given that patients cannot be randomized to different treatment regimens. Preclinical models offer the advantage of assessing individual chemotherapeutic agents and elucidating their specific molecular mechanisms. By employing preclinical models, researchers can also better regulate variables such as age, sex, environmental factors, type of cancer, treatment types, and comorbidities [see review by Seigers et al. (182)]. Several recent studies have demonstrated cognitive and neurobiologic deficits in mice treated with doxorubicin, cyclophosphamide, or cisplatin chemotherapy (183–188). Potential mechanisms of CRCI examined in these studies include sphingosine-1-phosphate receptor 1 activation, brain derived neurotrophic factor levels, apolipoprotein E4 genotype, and neuroinflammation [see review by Gibson and Monje (189) for potential mechanisms]. However, few studies have compared different chemotherapy regimens or utilized combination chemotherapies and therefore, significant work is still required in this area.

## Future directions

Biotyping is a nascent field that warrants replication of results across several dimensions, which is known as deep validation (72, 190). Deep validation steps including applying an existing biotype algorithm to new, independent data without reclustering to determine if biotype expression remains the same as in the original data, clustering independent data with a naïve algorithm to determine if biotypes can be reproduced, and extending cross-sectional biotypes to longitudinal data (72). These methods aim to determine whether the existence of biotypes in a particular condition is reliable, if the original biotyping solution is generalizable, and if biotypes can predict different disease trajectories.

Most biotyping studies to date have employed fMRI neuroimaging. However, neuroimaging it is not currently standard practice for breast or other non-CNS cancers, it is costly and associated with contraindications such as electronic or magnetic biomedical implants, magnetic orthodontia, and claustrophobia, among others. Neuronal activity is associated with increased oxygen delivery and the difference, or contrast between diamagnetic oxygenated hemoglobin and paramagnetic deoxygenated hemoglobin is measured by MRI. This is known as the blood-oxygen-level-dependent (BOLD) contrast and is the standard method used in functional MRI (191). There are other non-invasive technologies that are used to measure brain activity including electroencephalogram and magnetoencephalography. However, functional near-infrared spectroscopy (fNIRS) is the most similar available technology to fMRI. FNIRS also measures the BOLD contrast but does so using near-infrared light. Whereas most biological tissues are transparent to near-infrared light between 700 and 900 nanometers, hemoglobin absorbs and scatters near-infrared light in this range. Oxygenated and deoxygenated hemoglobin absorb near-infrared light at different wavelengths, which constitutes the BOLD contrast for fNIRS measurement (192, 193). FNIRS is highly tolerant to motion and has no environmental restrictions, contraindications or known risks (194).

The primary limitations of fNIRS are that it can only measure cortical tissue and cannot penetrate to subcortical structures (194) and most commercially available fNIRS devices only measure frontal regions. However, it is important to point out that prefrontal cortex is involved in most brain functions (195–197). Compared to chemotherapy naïve patients, chemotherapy-treated patients with breast cancer consistently show significantly changed prefrontal structure and function (74, 79, 82, 85, 198–201). Altered prefrontal regions are among the most predictive of long-term cognitive status in patients with breast cancer (147, 148). Further, in patients with breast cancer, lower prefrontal-executive function is the single best predictor of medication non-adherence (202) and has been associated with decreased quality of life following chemotherapy treatment (203). Thus, prefrontal fNIRS has significant potential as a proxy for whole brain fMRI in the study of CRCI that could be more easily used in-clinic. Surprisingly, no studies to date have applied fNIRS to the study of CRCI.

The correspondence between liquid and neuroimaging biomarkers is another avenue of research for discovering potential proxy technologies that support biotyping. Several lines of evidence suggest that cancer and its therapies accelerate aging processes (77, 86, 204–206). Individuals with age-related neurodegeneration and related cognitive decline tend to demonstrate greater amyloid-beta peptide accumulation and tau hyperphosphorylation compared to cognitively normal individuals (207). Tau and amyloid-beta can be measured from serum or plasma using the commercially available, single-molecule array (SIMOA) assay (208–210). Several studies have demonstrated that blood-based measurements of amyloid-beta or tau using SIMOA are associated with age-related neurogenerative conditions (211–214) as well as other neurologic conditions (215, 216). We uniquely demonstrated that blood-based amyloid beta and tau are highly predictive of cognitive functioning in breast cancer survivors, in combination with age, cytokines, and anthropomorphic measures (217).

Inflammatory biomarkers have long been of interest to researchers studying CRCI. Objective measures of CRCI have been shown to correlate with some inflammatory cytokines (e.g., IL-1β, IL-6, TNF-α, CRP), other proteins (e.g., GM-CSF), neurodegenerative markers (e.g., Aβ − 42, Aβ − 40, tau), and methylation ratios (42). Subjective measures of CRCI have demonstrated both significant and nonsignificant correlations with cytokines (IL-4, IL-1β, IL-6, MCP-1, IGF-1) and no significant correlations with TNF-alpha or CRP (42), suggesting perhaps more complex and inconsistent relationships between inflammatory biomarkers and CRCI.

Circadian biomarkers that can be measured objectively with saliva and blood or biorhythms (e.g., actigraphy) and are implicated in numerous side effects seen in cancer including CRCI. Similarly, to inflammatory biomarkers, data on circadian markers and CRCI relationships have been inconsistent and additionally quite limited in cancer research. For example, a recent study by Ancoli-Israel and colleagues showed that disrupted circadian rhythms were associated with reductions in objectively measured neurocognitive function in their longitudinal study of women with breast cancer (218). However, other studies failed to find any association between circadian rhythmicity and cognition in women with metastatic breast cancer (219). We suggest more research is warranted on the relationships between liquid and rhythm biomarkers in CRCI.

Neurofilament light, NF-L, a marker of neuroaxonal injury, is another protein that has received recent attention in relation to neurodegenerative diseases, including Alzheimer’s dementia (220). Increased blood concentration of NF-L have also been detected in person with frontotemporal dementia (221), Huntington’s disease (222), and Parkinson’s disease (223), suggesting that NF-L may be a biomarker of neurodegenerative processes in general. Blood based assays using SIMOA to detect NF-L are commercially available but no studies of NF-L and CRCI have been conducted to date.

Another emerging biomarker of cognitive decline is known as neuron derived exosomes (NDE). Exosomes are extracellular vesicles that play an integral role in intracellular communication by conveying materials such as RNA and proteins between cells (224). Exosomes are released by a most cell types, including neurons, and can be detected in blood, saliva, and urine (225, 226). NDE’s appear to contribute to cognitive impairment by transporting toxins during pathological conditions (224). This is evidenced by findings of increased NDE levels in individuals with neurodegenerative syndromes (227, 228). Koh et al. provided a comprehensive overview of NDEs as potential biomarkers of CRCI (229).

CRCI research initiatives, including many funding opportunities, have emphasized the need for longitudinal studies as they determine patterns of individual cognitive change which can be more sensitive than a single static cognitive assessment. Longitudinal studies can also provide insight regarding the separate and combined effects of different anti-cancer treatments on cognitive function. However, large cross-sectional studies should not be neglected or discounted as they help establish reliable syndrome phenotypes (230) and are necessary for the initial steps in biotyping. Large multi-site, national studies or pooled studies are needed to validate neuroimaging biomarkers and biotypes of CRCI. Data sharing and pooling can be facilitated by online databases such as EBRAINS,^1^ the Human Connectome Project,^2^ and LONI,^3^ among others.

Finally, most CRCI studies to date have focused on breast cancer although growing evidence indicates cognitive impairment across cancer types (231). A critical component of future research will be the ability to demonstrate that biotypes can be applied to other cancer diagnoses to determine what CRCI is, agnostic to disease. Further research is required to examine biomarkers of CRCI across different cancer types.

## Conclusion

CRCI is a prevalent condition that is not fully understood and, as a result, not diagnosed accurately, limiting treatment trials and implementation. Advanced statistical methodology, such as growth mixture modeling, allows for determination of latent subgroups using self-report and longitudinal data, offering significantly more precision in understanding cognitive trajectories and outcomes. However, the main limitation of symptom-driven subtypes classification remains lack of biological data. Neuroimaging can be used to identify brain biomarkers both individually and in clusters to define biological subgroups to diagnosis and predict outcomes. Like symptom-derived subtypes, biotyping accounts for variability in patient functioning by evaluating brain function to define the best taxonomy for patients but does so by utilizing the mechanistic information required for precision medicine.

## Author contributions

SK: conception or design. SK, AH, SP, OP, and MW: acquisition, analysis, or interpretation of data, and drafting and revising the work. All authors contributed to the article and approved the submitted version.

## Funding

This work was supported by funding from the National Institutes of Health (1R01CA226080, 2R01CA172145).

## Conflict of interest

The authors declare that the research was conducted in the absence of any commercial or financial relationships that could be construed as a potential conflict of interest.

The handling editor NS declared a past co-authorship with the author SK.

## Publisher’s note

All claims expressed in this article are solely those of the authors and do not necessarily represent those of their affiliated organizations, or those of the publisher, the editors and the reviewers. Any product that may be evaluated in this article, or claim that may be made by its manufacturer, is not guaranteed or endorsed by the publisher.

## Footnotes

^1^ https://www.ebrains.eu

^2^ https://www.humanconnectome.org

^3^ https://ida.loni.usc.edu/login.jsp
